# The Colposcopic Atlas of Schistosomiasis in the Lower Female Genital Tract Based on Studies in Malawi, Zimbabwe, Madagascar and South Africa

**DOI:** 10.1371/journal.pntd.0003229

**Published:** 2014-11-20

**Authors:** Hanne M. Norseth, Patricia D. Ndhlovu, Elisabeth Kleppa, Bodo S. Randrianasolo, Peter M. Jourdan, Borghild Roald, Sigve D. Holmen, Svein G. Gundersen, Jayanthilall Bagratee, Mathias Onsrud, Eyrun F. Kjetland

**Affiliations:** 1 Norwegian Centre for Imported and Tropical Diseases, Department of Infectious Diseases, Oslo University Hospital Ullevaal, Oslo, Norway; 2 University of Oslo, Oslo, Norway; 3 Imperial College London, London, United Kingdom; 4 Institut Pasteur de Madagascar, Antananarivo, Madagascar; 5 Center for Paediatric and Pregnancy Related Pathology, Department of Pathology, Oslo University Hospital, Oslo, Norway; 6 Research Department, Sorlandet Hospital HF, Kristiansand, Norway; 7 Department for Global Development and Planning, Institute for Development Studies, University of Agder, Kristiansand, Norway; 8 Discipline of Obstetrics and Gynaecology, School of Clinical Medicine, Nelson R. Mandela School of Medicine, University of KwaZulu-Natal, Durban, South Africa; 9 Department of Gynaecology, Oslo University Hospital Ullevaal, Oslo, Norway; 10 Discipline of Public Health Medicine, Nelson R. Mandela School of Medicine, College of Health Sciences, University of KwaZulu-Natal, Durban, South Africa; Weill Cornell Medical College, United States of America

## Abstract

**Background:**

*Schistosoma (S.) haematobium* is a neglected tropical disease which may affect any part of the genital tract in women. Female genital schistosomiasis (FGS) may cause abnormal vaginal discharge, contact bleeding, genital tumours, ectopic pregnancies and increased susceptibility to HIV. Symptoms may mimic those typical of sexually transmitted infections (STIs) and women with genital schistosomiasis may be incorrectly diagnosed. An expert consensus meeting suggested that the following findings by visual inspection should serve as proxy indicators for the diagnosis of schistosomiasis of the lower genital tract in women from *S. haematobium* endemic areas: sandy patches appearing as (1) single or clustered grains or (2) sandy patches appearing as homogenous, yellow areas, or (3) rubbery papules. In this atlas we aim to provide an overview of the genital mucosal manifestations of schistosomiasis in women.

**Methodology/Principal findings:**

Photocolposcopic images were captured from women, between 1994 and 2012 in four different study sites endemic for *S. haematobium* in Malawi, Zimbabwe, South Africa and Madagascar. Images and specimens were sampled from sexually active women between 15 and 49 years of age. Colposcopic images of other diseases are included for differential diagnostic purposes.

**Significance:**

This is the first atlas to present the clinical manifestations of schistosomiasis in the lower female genital tract. It will be freely available for online use, downloadable as a presentation and for print. It could be used for training purposes, further research, and in clinical practice.

## Author Summary

Female genital schistosomiasis commonly remains undiagnosed due to its unacknowledged clinical manifestations. Millions of women in endemic areas are infected, and many suffer from abnormal vaginal discharge, contact bleeding, genital tumours, ectopic pregnancies, and an increased susceptibility to HIV. *S*andy patches and rubbery papules identified by visual inspection may serve as indicators for the diagnosis of genital schistosomiasis. However, text books do not contain this information and it is not taught in medical school or to nurses serving these patients in endemic areas. The aim of this Atlas is to present the photocolposcopic manifestations of schistosomiasis in the lower female genital tract. Photocolposcopic images were captured from women between 15 and 49 years of age, between 1994 and 2012 in four different sites endemic for *S. haematobium* in Malawi, Zimbabwe, South Africa and Madagascar. This is the first atlas to present the clinical manifestations of schistosomiasis. Two types of sandy patches, abnormal blood vessels and rubbery papules are shown, as well as differential diagnoses. PloS NTDs makes it possible for all to access this information.

## Introduction

The objective of this paper is to provide an overview of gynaecological lesions due to *S. haematobium* in the lower female genital tract for clinicians, researchers and health professionals in training. The material is based on investigations by the group in the last 20 years in, Malawi, Zimbabwe, Madagascar and South Africa.

### The extent of the problem

Urogenital schistosomiasis is most commonly caused by *S. haematobium*; however, cases of urogenital infections with other schistosome species have been reported. *S. haematobium* is particularly common in Africa, but may also occur in the Middle East. Previously, *S. haematobium* infection has been referred to as urinary schistosomiasis [1]. With the new knowledge of the severity and prevalence of genital tract affliction, in both females and males, the World Health Organization (WHO) recommends that the disease should be called urogenital schistosomiasis [1].

### Epidemiology and clinical consequences

Female genital schistosomiasis affects at least 16 million women in endemic areas, and may cause abnormal vaginal discharge, contact bleeding, ectopic pregnancy, and possibly an increased susceptibility to HIV [2]–[8]. Several of these symptoms and signs may be caused by the immunologic reaction to schistosome eggs in the tissues.

**Figure 1:**
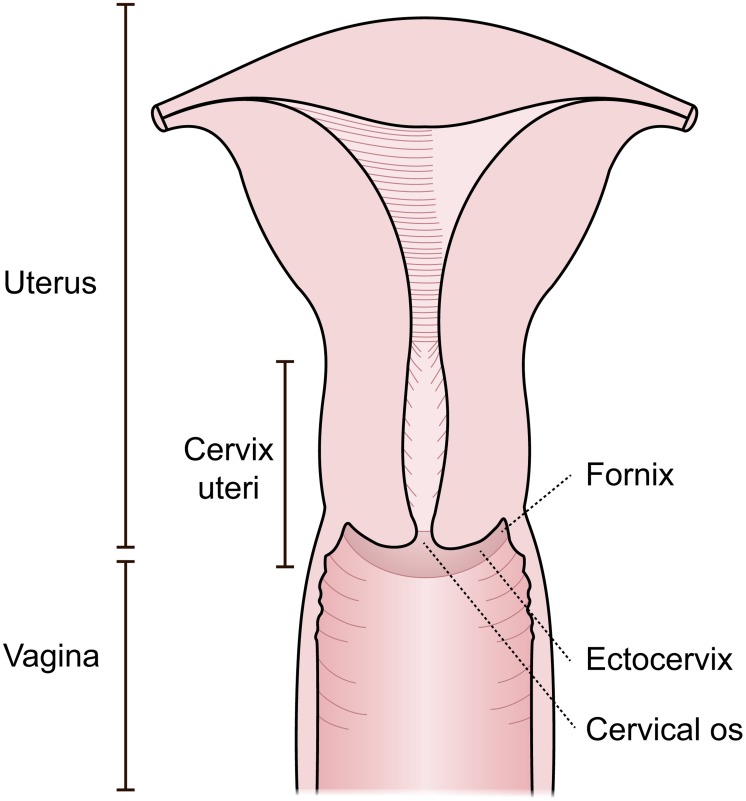
Possible sites for disease by *S. haematobium* in women. Manifestations in the cervix and vagina are seen with a colposcope, but ova seem to be evenly distributed in all parts of the genital tract [12].

The lesions caused by *S. haematobium* in the lower genital tract may be identified by the colposcopic examination of the cervix, vagina and vulva (Figure 1), and are most commonly found on the cervix [9]–[11]. Autopsy studies indicate that *S. haematobium* ova are found in any location of the female genital tract [12]. Lesions may be seen as sandy patches, abnormal mucosal blood vessels and rubbery papules [6], [7], [13], [14] (Randrianasolo, in progress). These focalized lesions are difficult to detect by visual inspection.

### Early problem in girls

**Figure 2:**
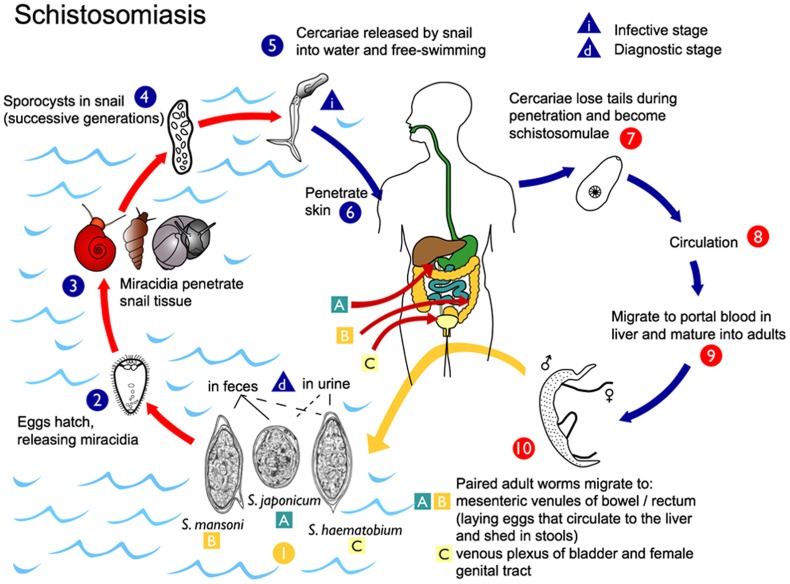
Schistosomiasis life cycle. The most common species found to be pathogenic to humans; and *Schistosoma (S.) haematobium, S. mansoni, S. japonicum* [73]. Eggs are excreted through faeces, urine and possibly through vaginal discharge from infected individuals, may hatch if they come in contact with water, releasing miracidia that infect fresh water snail hosts where they multiply, producing free-swimming cercariae that eventually may penetrate the skin of human hosts. The cercariae mature in the portal vein and migrate to venules draining the intestines, or the urinary and genital tracts, where they may deposit up to 300 eggs every day. Some of these eggs will be trapped in the tissues inducing a localised host response, while others will penetrate the vessel wall and the mucosa of the intestines, the bladder or the genitals, subsequently excreted in faeces, urine or vaginal discharge into fresh water in order to continue the parasite life cycle. This figure shows the venous plexus of the bladder only; however, the venous plexi surrounding the genital tract is also affected. (Source: CDC-DPDx, Atlanta, United States).

Most girls and women living in endemic areas acquire schistosomiasis during childhood when in contact with schistosome infested water, for recreational, domestic or other purposes as portrayed in Figure 2 [15], [16]. High worm loads acquired after years of water contact are more likely to create clinical problems, but short exposure may also have serious consequences, such as pain or salpingitis and schistosoma-induced non-malignant tumours [17]–[21].

Genital schistosomiasis has not been systematically inspected in girls; however, some papers suggest that the infection may cause manifestations already at an early stage in life [22], [23]. Gynaecological examinations are seldom performed in young girls prior to the first sexual intercourse, and hence case reports from girls are mostly reports of the vulvar schistosomal lesions [24]–[33]. A few cases of vaginal and cervical schistosomiasis have been reported in young women [34]. Furthermore, there are reports of decreased fertility and arrested development of corpora lutea in animal models, and of stunting and late pubertal development in humans, suggesting that schistosomiasis also may cause hormonal disturbances [24], [35]–[40].

Systematic investigations of urinary schistosomiasis have shown that urinary tract lesions in children resolve within two to six months post-treatment, whereas lesions in adults are resistant to anti-schistosomal treatment [41]–[53]. The effect of early treatment of genital schistosomiasis needs to be explored.

### Male genital schistosomiasis

There have been a number of reports of haematospermia in men with genital schistosomiasis, even in men with negative urines [54], [55]. The issue will not be discussed in detail but briefly two Madagascan studies on men report *S. haematobium* ova in semen and concomitant haematospermia, increased leukocyte counts and cytokine levels [56]. Dually infected men, with schistosomiasis and HIV, have been hypothesised to pose a risk of HIV transmission to their partners, and their semen could contaminate female genital specimens.

### Diagnostic approaches for schistosomiasis in the lower female genital tract

#### Visual examination

An expert meeting in 2010 suggested that in patients from *S. haematobium* endemic areas, one or more of the following three clinical findings are adequate for a clinical diagnosis of schistosomiasis in the lower female genital tract [12]: Sandy patches appearing as (1) single or clustered grains or (2) sandy patches appearing as homogenous, yellow areas or (3) rubbery papules (Text S1). All three types of lesions may be found together with abnormal blood vessels, all aceto-white reaction negative, and stain as normal tissue when applying Lugol's iodine solution [12]. Female genital schistosomiasis (FGS) is therefore distinct from lesions associated with neoplasia.

#### Investigations in urine

**Figure 3:**
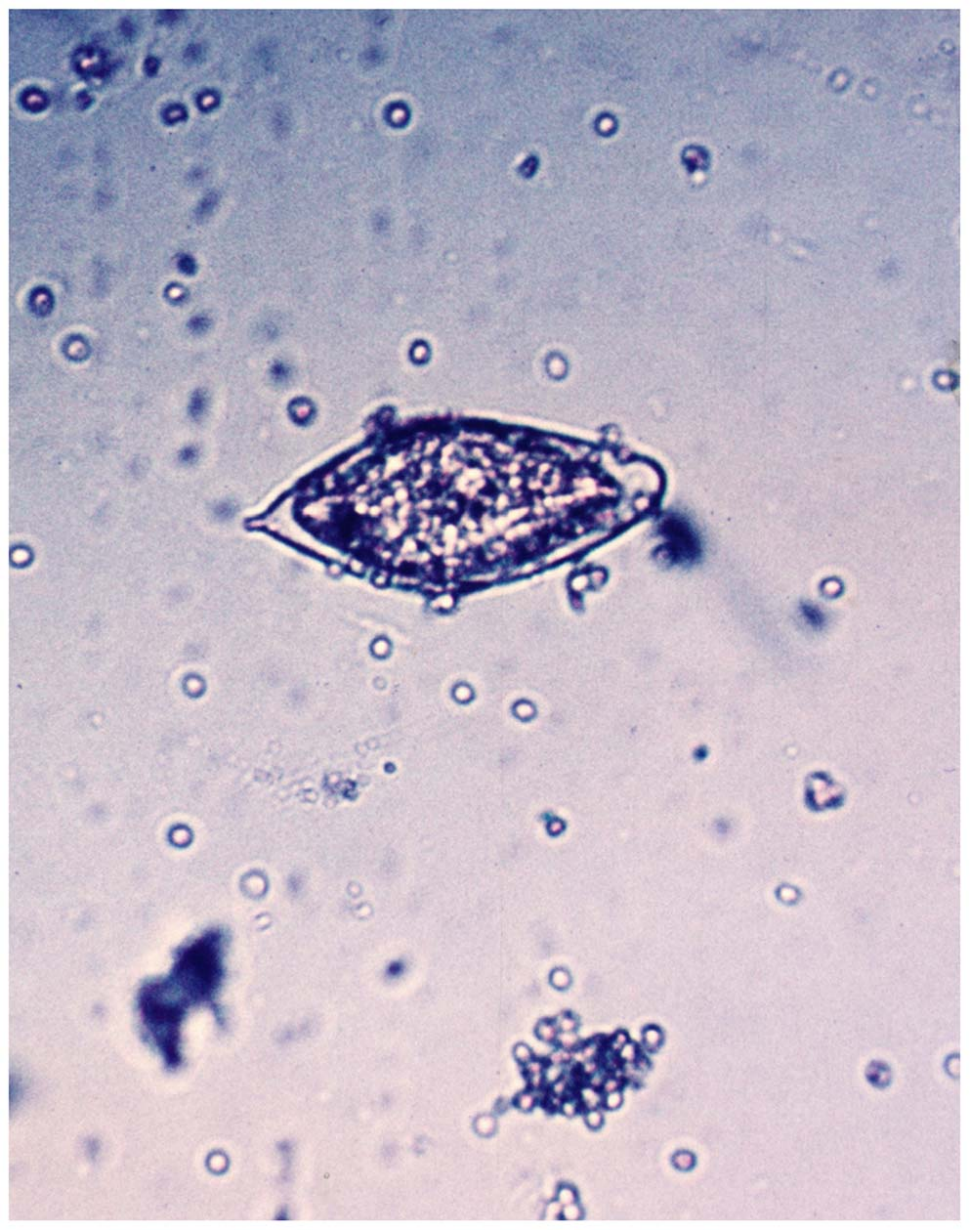
*S. haematobium* ovum as seen in urine microscopy.

Some studies indicate that less than 60% of women with FGS excrete schistosome ova in the urine, hence urine analysis alone is not adequate for an appropriate diagnosis [6], [57]. Testing for urinary *S. haematobium* infection, may be done by microscopy of the sediment following centrifugation of 10 mL of urine (Figure 3), or following urine filtration for the ova. Where there is no centrifuge, or the procedure cannot be performed for other reasons, the urine may stand in a conical sample container for some hours, before examination of the sediment. Several urine samples should be investigated and in low-intensity infections it may be necessary to explore large volumes over several days in order to detect infection [12]. Eggs hatch at room temperature, but storing the urine in a fridge or adding formalin can prevent this.

#### Biopsy sample taken of genital lesions

Where it is clinically and ethically feasible, a bedside crushed biopsy taken from a suspected lesion has been purported to be one of the most sensitive diagnostic methods for FGS [58], [59]. This method does, however, have some disadvantages. Firstly, it precludes the possibility for histological analyses. Secondly, the biopsy punch is a crude sampling method of the small schistosome lesions and may fail to include the eggs [60]. Furthermore, this method has been suggested to pose an increased risk of HIV transmission for the patient and her partner until the inflicted mucosal wound has healed [12]. Lastly, ova may be found in clinically normal tissue [30], [60].

#### Schistosome polymerase chain reaction (PCR)

Lesions may be chronic in adults and can persist in the absence of live ova or worms [16]. Old lesions may still be present and live eggs may be found in other locations not detected by PCR [12]. *Schistosoma* real-time PCR may be run in vaginal lavage and biopsy material [61] (Randrianasolo, in progress). The ova with miracidia DNA may live for some weeks and the worm can continue to lay eggs for a lifetime [62]. The average life span of a worm is five years, but occasionally live worms have been found in humans up to 30 years after exposure. A positive schistosoma PCR result may indicate schistosomal disease in the female genital tract, or may be caused by ova contamination from urine or semen.

#### Cervical cytology

**Figure 4:**
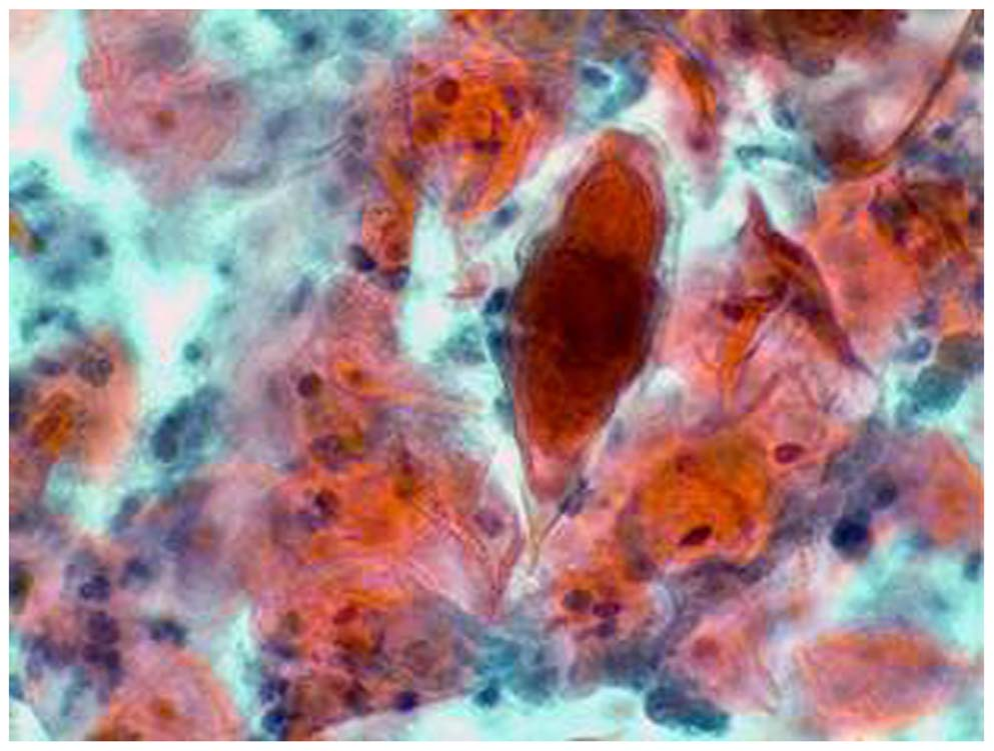
Pap smear. *S. haematobium* ovum with terminal spine.

Papanicolaou (Pap) smears have been shown to have a low sensitivity for the diagnosis of schistosomiasis in the female genital tract [6], [59], and should not be used to preclude genital schistosomiasis (Figure 4). However, results from Madagascar indicate that this test may be useful in some areas (Randrianasolo, in progress). A positive result may also be due to contamination from urine or semen.

#### Other tests

Serology, Circulating anodic antigen (CAA), Eosinophil Cationic Protein (ECP) and Eosinophil Protein-X (EPX, same as eosinophil derived neurotoxin, EDN) do not provide information of the location of the clinical problem. Serological tests range from 70% in sensitivity and will very often remain positive after treatment. CAA may indicate the presence of a live worm, but will be negative if the parasites are dead and the ova calcified. The eosinophil products rely on the host's eosinophil reaction to the ova. In chronic disease, this is often not present. Furthermore, eosinophil tests may become positive in other diseases, such as asthma [63].

## Methods

### Ethical approval in the four study sites

In Zimbabwe, the Provincial and District Medical Directors, the village headman and village meetings gave their permission to conduct the study. Ethical approval was given by the Medical Research Council of Zimbabwe and by the ethical committee of the Special Programme for Research and Training in Tropical Diseases Research, UNDP/WB/WHO. While in Malawi, ethical approval was given by the Medical Ethical Committee of Malawi, Ministry of Health and Environmental Affairs 1993 and by UNDP/WB/WHO TDR. In South Africa, four ethics' committees granted permission to perform the study; Biomedical Research Ethics Administration, University of KwaZulu-Natal (KZN), Department of Health, Pietermaritzburg, KZN, Regional Ethics Committee (REK) Eastern Norway, and the European Group on Ethics in Science and New Technologies 2011. The Departments of Health and Education in KwaZulu-Natal gave local permission. In Madagascar, ethical permission was obtained from the Committee of Ethics at the Ministry of Health in Madagascar.

Study information was provided to the study populations in the local languages. Informed oral or written consent was obtained. Oral informed consent was obtained in Malawi some hours prior to the investigations. It was done in accordance with the ethics approval from the Ethical committee of the Special Program for Research and Training in Tropical Diseases Research/World Bank/World Health Organization in1993 and documented on the interview forms as was general practice at the time and location. In the three other study sites, written informed consent procedures were performed. Furthermore in each of the study sites the woman was asked before each step if she was willing to participate. Following consent, all women who fulfilled the inclusion criteria were offered gynaecological examination (Table 1). Consent was also re-ascertained by the physician before each step of the investigation. Treatment and follow-up for schistosomiasis, sexually transmitted infections (STIs), cancer and other conditions were given in all sites.

**Table 1:** Specific facts in four recruitment sites.

Study site (total number of women investigated)	Age range	Mean age (years)	Urinary *S. haematobium* a	Inclusion criteria age, non-virgins, not pregnant	Published
South Africa (n = 900)^b^^,^ c ^,^ d	16–23	18	Endemic area	All pupils in high schools invited	In progress
Madagascar (n = 118)^b^	15–35	20	Known low and high-endemic villages	All in village screened (79 positive and 39 negative)	In progress
Zimbabwe (n = 527)^c^	20–49	33	Endemic area	All in four villages invited	[6], [61]
Malawi (n = 52)^e^	15–49	22	All positive	All in outpatient department	[58], [60], [65]

a The presence of a single terminal-spined ovum gave a positive diagnosis *S. haematobium*.

b Olympus OSC 500, Olympus America Inc., Center Valley, PA, USA and Olympus E420, 10.0 megapixels, Olympus America Inc. USA,

c Leisegang Photocolposcope, Script-O-Flash, Germany, Magnifications 7.5; 15; 30.

d Canon EOS mounted on colposcope.

e Leisegang Stereo-photocolposcope.

### Study populations

**Figure 5:**
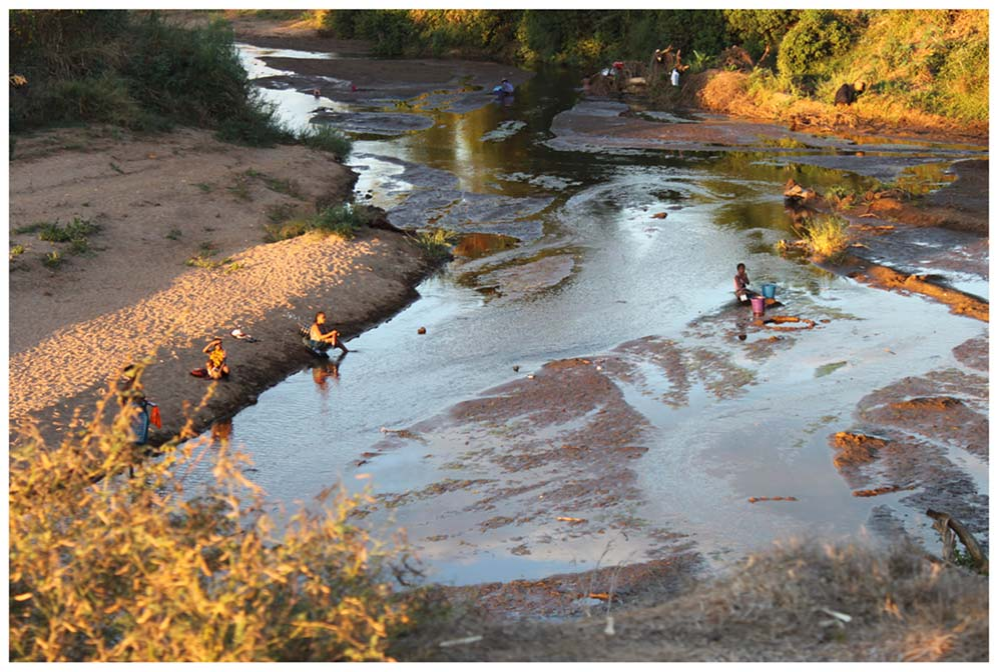
A typical transmission site. River water is used for personal, household, animal husbandry and recreational purposes. Even where there are taps the queues are often long. Water that is not for drinking purposes is acquired from fresh water bodies as the one shown in the photo.

Table 1 shows the selection criteria of consenting females in four different rural study sites endemic for *S. haematobium* in Malawi, Zimbabwe, South Africa and Madagascar between 1994 and 2012. All areas were low-endemic for *S. mansoni*. In all sites, except for Madagascar, some women had access to safe water sources; however, rivers were commonly used or had been used for laundry, playing and bathing (Figure 5). Patients were pre-menopausal and aged 15 to 49 years of age, the mean age varied according to study protocol (Table 1).

### Clinical examination

**Figure 6:**
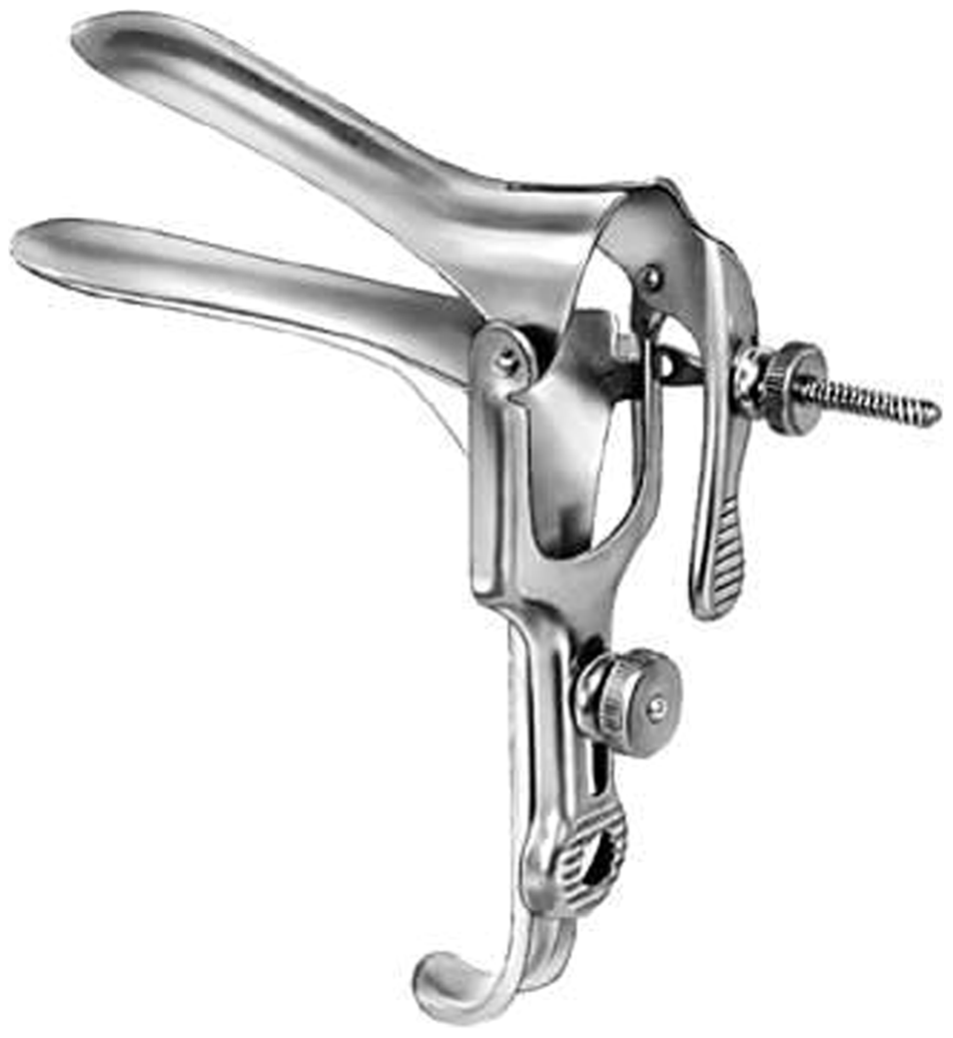
Different speculums. In our experience speculum A was the only speculum that allowed rotation for full inspection if the vaginal walls. The others caused discomfort. Disposable speculums are expensive and often do not hold well rotated for the inspection of the anterior and posterior vaginal walls.

After insertion of a metal speculum (Malawi, Zimbabwe, South Africa, Figure 6) or a disposable plastic speculum (Madagascar) the gynaecological examination was performed in four steps: Cervicovaginal lavage; saline (5 ml or 10 ml as per protocol) was sprayed on the vaginal walls and cervix, drawn back into a syringe, and deposited into cryotubes. Thereafter, inspection of the mucosal surfaces was performed with the colposcope according to a predefined protocol, section by section. Mucosal abnormalities were documented. Then Pap smears were done in all consenting women. Lastly, anterior and posterior surfaces of the vaginal wall were inspected by rotating the speculum 90 degrees, and morbidity was documented. The inspection is only possible with a sturdy metal or a high quality plastic speculum (no sharp edges). In order to ensure that no contaminants (e.g. STIs, eggs or miracidia) were transferred, the metal speculums were autoclaved in all sites.

The homogeneous yellow sandy patches were defined as sandy looking areas with no visible grains when using the 15 times magnification setting on the colposcope [6]. The grains of the sandy patches are approximately 0.05 mm by 0.2 mm long, are shaped as minuscule rice grains, they may be single or in clusters of up to 300. The abnormal mucosal blood vessels in genital schistosomiasis were defined as pathological convoluted (cork-screw), reticular, circular and/or branched, uneven-calibered blood vessels [6].

### Photocolposcopic imaging and quality control

Approximately 4000 colposcopic images were captured and the images of the highest technical quality were chosen. The photocolposcopic equipment used in the respective study sites are given in Table 1. Eyepieces, lamps, bulbs, and surrounding light conditions were adjusted and more than 15 times magnification was often needed. The micro-meter focusing function was used continuously. For the review process in making this atlas printed, images had to be colour-proofed, balanced and converted to CMYK, using the colour profile of the printer (Text S1).

A panel of experts in tropical diseases, genital schistosomiasis and gynaecology reviewed the findings using a projector, a computer screen or a monitor. The screens or projectors were focused, light adjusted or contrasted and/or the screen tilted for optimal viewing. Only images with an adequate resolution for determining the diagnostic details were used.

### Visual diagnosis of *S. haematobium* infection in the lower female genital tract

The findings caused by *S. haematobium* infection in the lower female genital tract may be subtle and focal, and may be easily missed. FGS cannot be precluded without the systematic use of a colposcope viewing the entire mucosal surface, including the vaginal fornices. Rotating the speculum is necessary to view the posterior and anterior vaginal walls. Most importantly the patient must be given enough information, time and privacy to be completely relaxed during the examination.

## Results

### Sandy patches and rubbery papules

**Figure 7:**
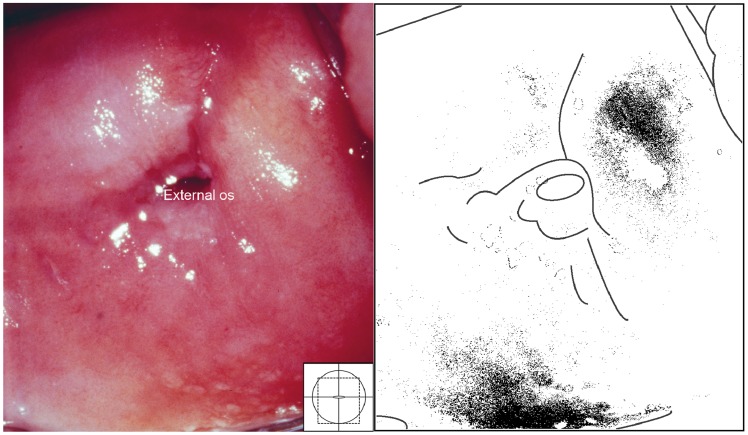
Homogeneous sandy patches. Sandy patches appearing as a homogenous, yellow area. There is also some white discharge at six o'clock. The colour analysis (black and white template) shows that the typical yellowish colour is found 1 to 2 o'clock and 6 o'clock (Holmen, submitted).

**Figure 8:**
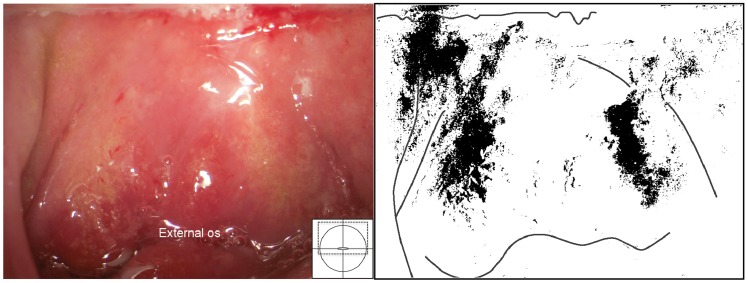
Grainy sandy patches and mucosal bleeding. Grainy sandy patches on the entire anterior lip of the cervix, on the endo- and ectocervix, into the anterior and lateral fornices. Note the different shades of yellow; some areas are bright yellow, whereas other areas are beige to white. Mucosal bleeding is seen in especially in the anterior fornix.

**Figure 9:**
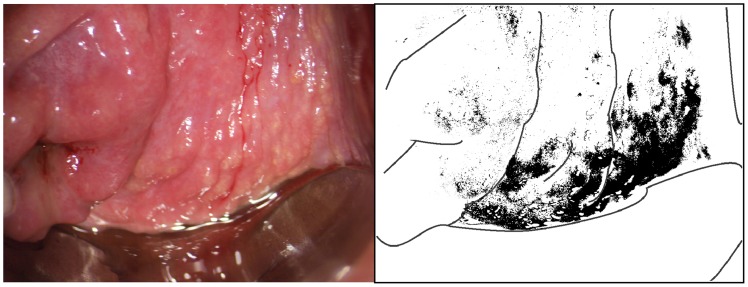
Grainy sandy patches on the vaginal wall. Clusters of grainy sandy patches and mucosal bleeding of the lateral and posterior vaginal walls. The vaginal mucosa looks hyperaemic, but no vessel structures are seen at this magnification.

**Figure 10:**
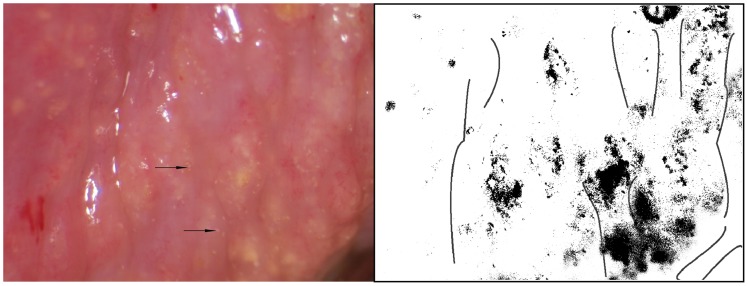
Sandy patches appearing as grains and homogenous, yellow areas of the vaginal wall. Enlarged section of a part of the vaginal wall in Figure 9. At this magnitude we see the single grains' (arrows point to some examples) characteristic rice-grain shape and colour. The entire surface has a mottled appearance. We also see homogenous yellow areas with embedded grains.

**Figure 11:**
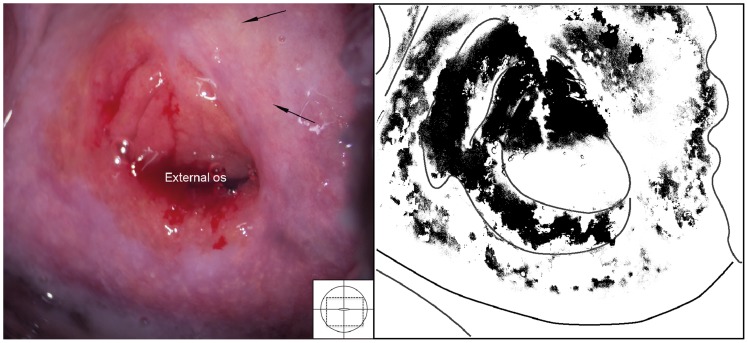
Sandy patches appearing as grains, homogenous, yellow areas, abnormal blood vessels and mucosal bleeding. The entire cervical surface is mottled by clusters of grains and some homogeneous yellow areas with single grains embedded (arrows point to some examples). The whole transformation zone looks yellow, possibly due to the extensive amount of ova. We also see mucosal bleeding from around the cervical os.

**Figure 12:**
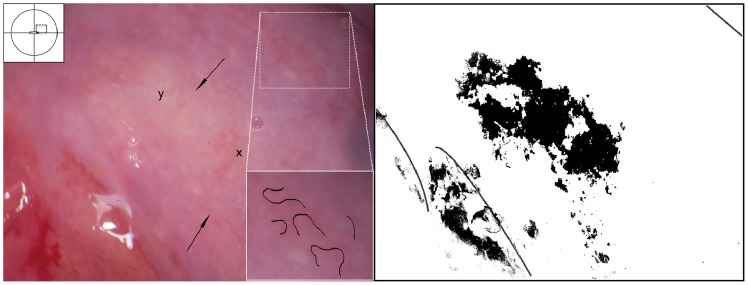
Grains embedded in a homogeneous yellow area. Enlarged section of a part of the ectocervix in Figure 11. Single grains (arrows point to some of them) are embedded in the homogeneous yellow area. The different shades of yellow in this lesions range from a dull, almost brownish colour (x) to a sharp, gold-like colour seen in the single grains (y). Not every person in our group was able to see these nuances. The colour analysis (black and white template) may assist (Holmen, submitted). The magnified insert shows the contours of the adjacent typical abnormal blood vessels.

**Figure 13:**
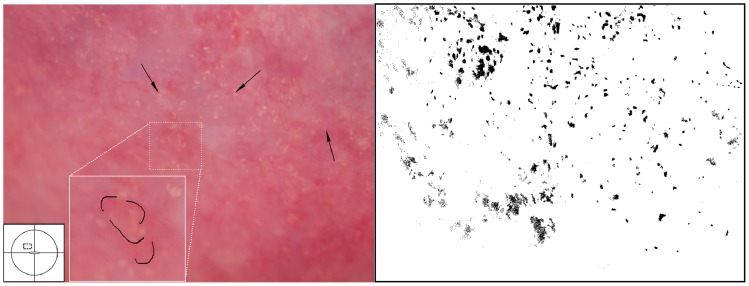
Grainy sandy patches and abnormal blood vessels. Enlarged section of a part of the ectocervix. Clusters of grains and single grains are spread over the ectocervical surface. The single grains (arrows point to some examples) are approximately 0.05 by 0.2 mm in size with a rice-grain shape and yellow colour. The grains are surrounded by a network of convoluted blood vessels. The insert shows the contours of the adjacent abnormal blood vessels.

**Figure 14:**
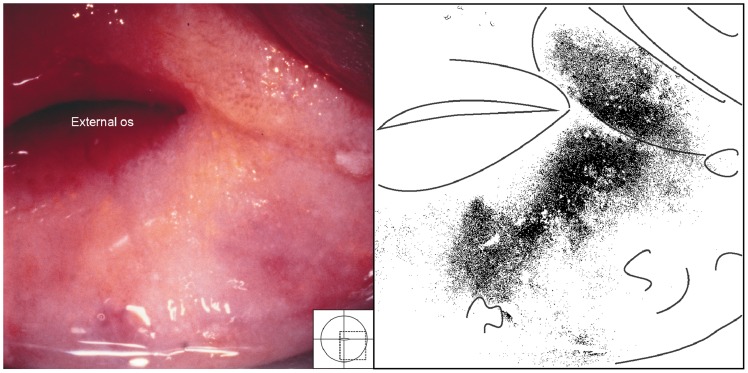
Sandy patch appearing as a homogenous, yellow area. The homogeneous yellow area can be seen as a yellow discolouring of the mucosa. A lesion like this can be very difficult to spot if one does not have the correct light source.

**Figure 15:**
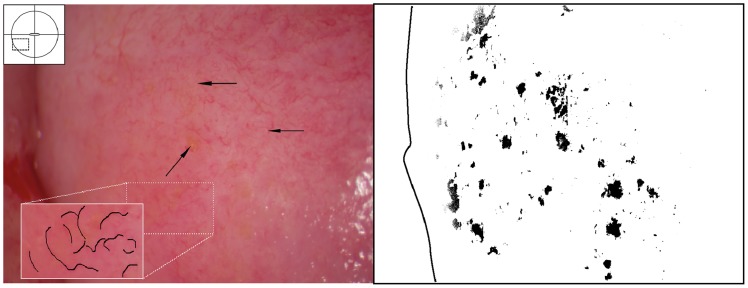
Sandy patches appearing as single grains and homogenous, yellow areas surrounded by abnormal blood vessels. Ectocervical mucosa with single grains (arrows point to some examples) scattered all over and surrounded by a network of abnormal blood vessels. When looking at this closely and from different angles, perhaps by tilting the computer monitor or adjusting the brightness level, one can see small areas with a yellow colour, representing homogeneous yellow areas. The insert shows the contours of the adjacent abnormal blood vessels.

Two types of sandy patches have been identified sandy patches appearing as (1) single or clustered grains or (2) sandy patches appearing as homogenous, yellow areas (Figure 7). The grains are deep or superficially situated in the mucosa, with a characteristic yellow, off-white or golden colour (Figure 8). The deeply situated grains merge into sub-mucosal plaque-like formations with uneven edges and shades of texture (Figure 9, 10). Sometimes the mucosa is mottled beneath the surface (Figures 11, 12). The mucosal surface over the deeply grained patches is smooth and grains are not moveable. The superficial grains have a distinct shape and colour (Figure 8). Grains can often be distinguished easily from each other even when they are clustered together. Occasionally, with a metal spatula, movable distinct minuscule crust-like superficial protrusions can be felt. These may cover the whole vaginal or cervical surface (Figure 8), but sometimes only one grain or a few individual grains are seen (Figure 13). The grainy and homogeneous sandy patches can be found concurrently (Figures 12, 14). They do not respect the squamo-columnar junction and they are not confined to the transformation zone. The sandy patches are often but not always accompanied by other lesion types such as abnormal blood vessels or general signs of inflammation, but are always aceto-white reaction negative (Figure 15). In some cases with clusters of grains, the mucosa is hyperaemic or inflamed (Figure 11). The mucosa is often fragile, and the surfaces may bleed on touch (contact bleeding).

**Figure 16:**
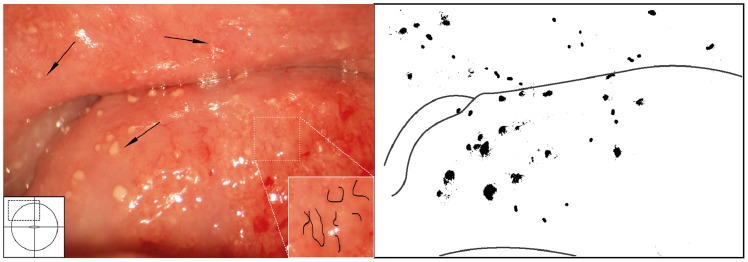
Rubbery papules and abnormal blood vessels. Rubbery papules and mucosal bleeding on the cervical surface and anterior fornix. Papules look like pustules but are firm like rubber, the diameters range between 0.3 to 1.2 millimetres. Near the papules are minute-spiral blood vessels (arrows point to some examples).

**Figure 17:**
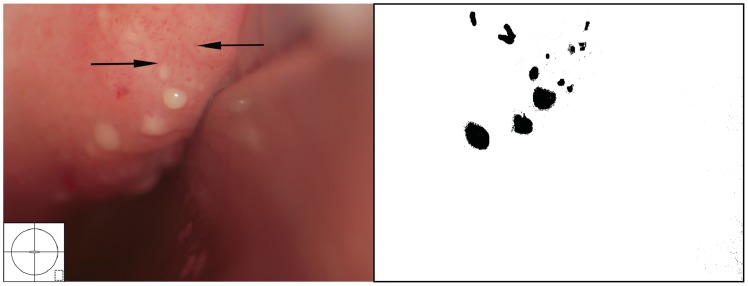
Rubbery papules. Enlarged section of the lesion in Figure 16. The black and white template shows that the colour of the rubbery papules is recognised by the colour analysis (Holmen, submitted). Near the papules are minute-spiral blood vessels (in red). Tilt the monitor to see more detail.

Rubbery papules were only found and documented in Madagascar (Figures 16, 17). The same clinician (EFK) was clinically active in all study sites. All images from the different study sites were re-reviewed to explore if the rubbery papules might have been overlooked during previous investigations. Not a single case was identified in the other locations. The rubbery papules are spheroid, pustuloid and firm (hence rubbery), beige papules in the cervicovaginal mucosa. The 0.3–1.2 mm papular lesions are easy to spot with the naked eye (Figure 16). They give the mucosa an irregular surface. The rubbery papules may stand alone, or be found concurrent with sandy patches. They are often surrounded by various degrees of vascularisation at their base (Figures 17); both abnormal blood vessels and mucosal bleeding may be seen.

### Histopathologic findings

**Figure 18:**
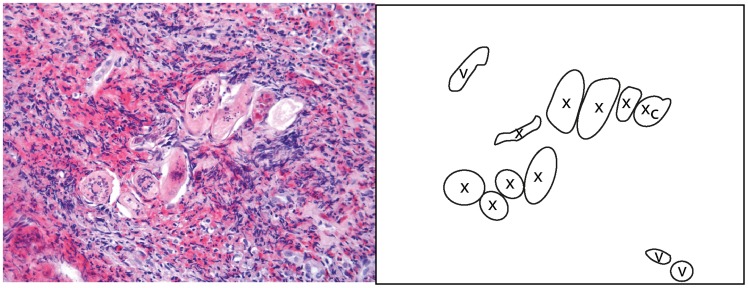
Histological correlate of a rubbery papule to **Figures 17** and **18**. In rubbery papules viable-looking (with intact structures) schistosome ova (x) are surrounded by intense eosinophilia.

**Figure 19:**
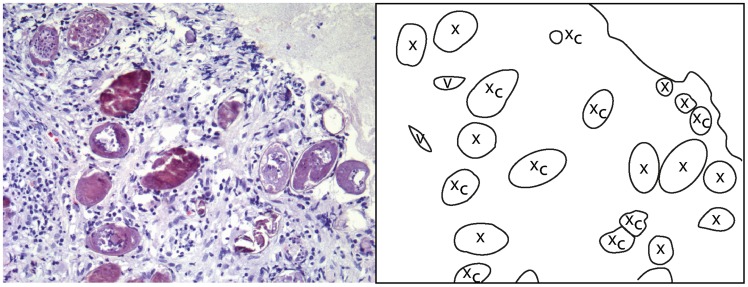
Histological correlate to the sandy patch in **Figure 9**. Numerous calcified (x_c_) and viable-looking ova (x) are seen in the stroma beneath the epithelium. Lymphocytes, eosinophils and immature fibroblasts surround the schistosome eggs.

Microscopic examination of the cervicovaginal schistosome lesions frequently reveals viable and/or dead schistosome eggs in the stroma (Figure 18, 19). No adult worms were identified in this material; maybe due to the biopsies being small and samples being superficial.

### Differential diagnoses

#### Cervical intraepithelial neoplasia (CIN)

**Figure 20:**
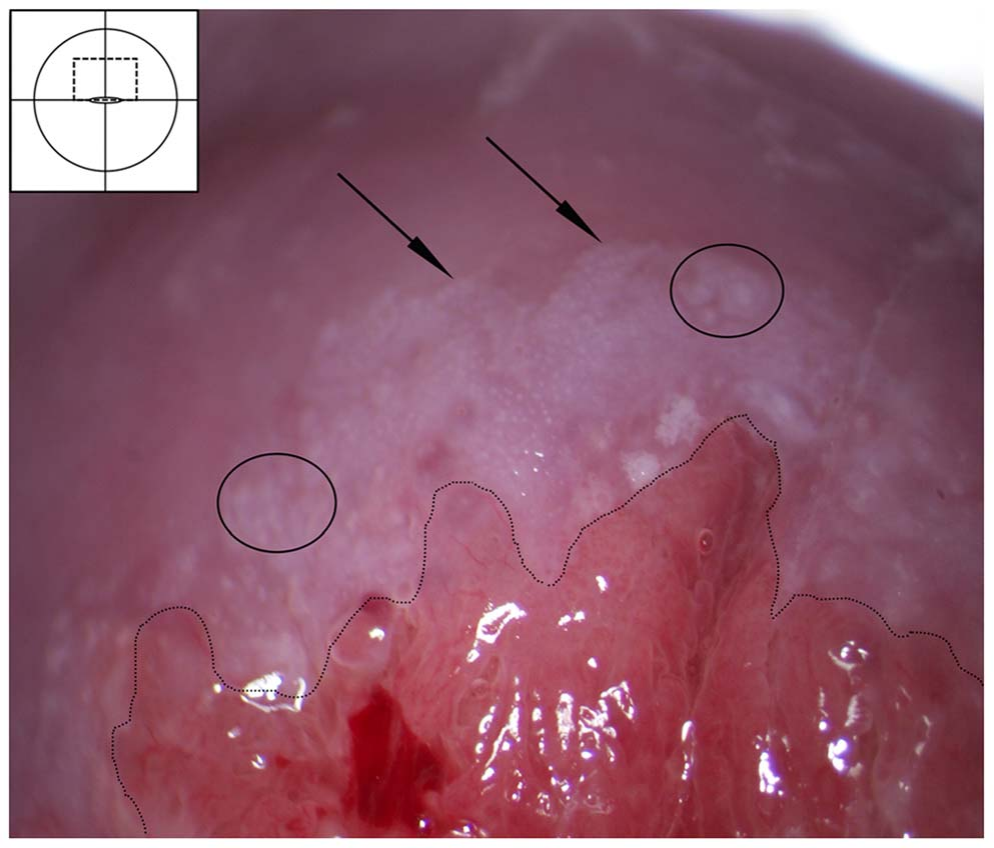
Cervical intraepithelial neoplasia stage I–II after application of acetic acid. Aceto-white lesion in the transformation zone abutting the squamo-columnar junction (dashed line). The white area is dense and has feathery margins (arrows), possibly with some mosaic pattern (ovals). This finding probably represents cervical intra-epithelial neoplasia (CIN) stage one to two. CIN refers to the premalignant neoplastic changes taking place in the squamous epithelium in the transformation zone of the cervix before the possible development of cervical squamous carcinoma. These changes can be divided into three groups based on the proportion of epithelium thickness involved in the dysplastic process. Early stages of CIN may be confused with homogenous yellow areas of the sandy patches, and late stages may involve some of the same vessel patterns that can be seen in schistosome lesions [6]. However, the schistosome lesions are not aceto positive, and they are not confined to the transformation zone.

**Figure 21:**
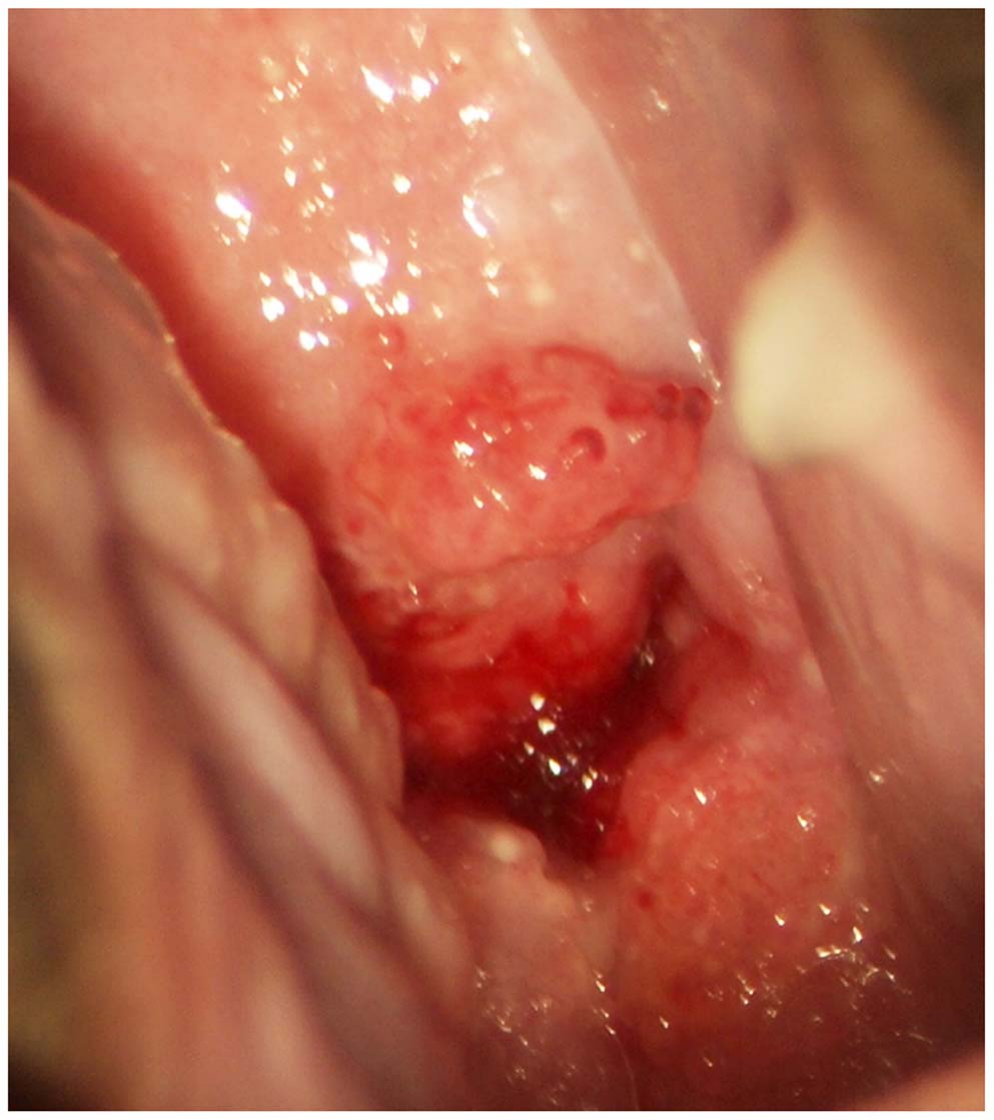
Malignant looking lesion. Severe cervicitis caused by schistosomiasis. Hysterectomy and cone biopsies have been performed in lesions like this due to lack of pathology services and ignorance [6].

In contrast to cervicovaginal schistosomiasis, CIN causes an aceto-white positive reaction. CIN is located within the transformation zone whereas schistosomiasis may be located anywhere in the genital mucosa. CIN and schistosomiasis have variable margins, surface contours and vascular patterns. The low-grade CIN lesions are characterised by feathery margins (“geographic”) and smooth surfaces (Figure 20). This may occasionally be seen in homogenous sandy patches, but these lesions are always aceto-white reaction negative. The high-grade CIN lesions are clearly demarcated (Figure 21), often with raised margins. The dense and varying colour intensity and irregular surface contours in high-grade CIN may potentially be mistaken for schistosomiasis. The high-grade lesions are often associated with different vascular patterns, such as mosaics or coarse punctation, whereas abnormal mucosal blood vessels associated with schistosomiasis often portray a larger reticulated pattern (Figure 15).

#### Cervical cancer

The finding of abnormal blood vessels should always raise the suspicion of malignancy (Figure 21). The mucosal blood vessels in schistosomiasis may be very difficult to distinguish from those of cancer. Invasive cancer is a solitary lesion starting in the transformation zone that can be proliferative exophytic or ulcerative with contact bleeding and foul smelling discharge as typical symptoms. Areas with CIN are frequently found around an early stage malignant tumour. The definitive diagnosis of cancer must be made with a biopsy and histological examination.

#### Flat condylomas caused by human papillomavirus

Aceto-white reaction positive lesions with sharply demarcated, elevated and cauliflower-like surfaces, mostly multiple and located outside the TZ. To date schistosomiasis has not been found to be associated with human papillomavirus (HPV) infection; however, *S. haematobium* ova have been found inside condylomas that have not been explored for HPV aetiology.

#### Nabothian cysts

**Figure 22:**
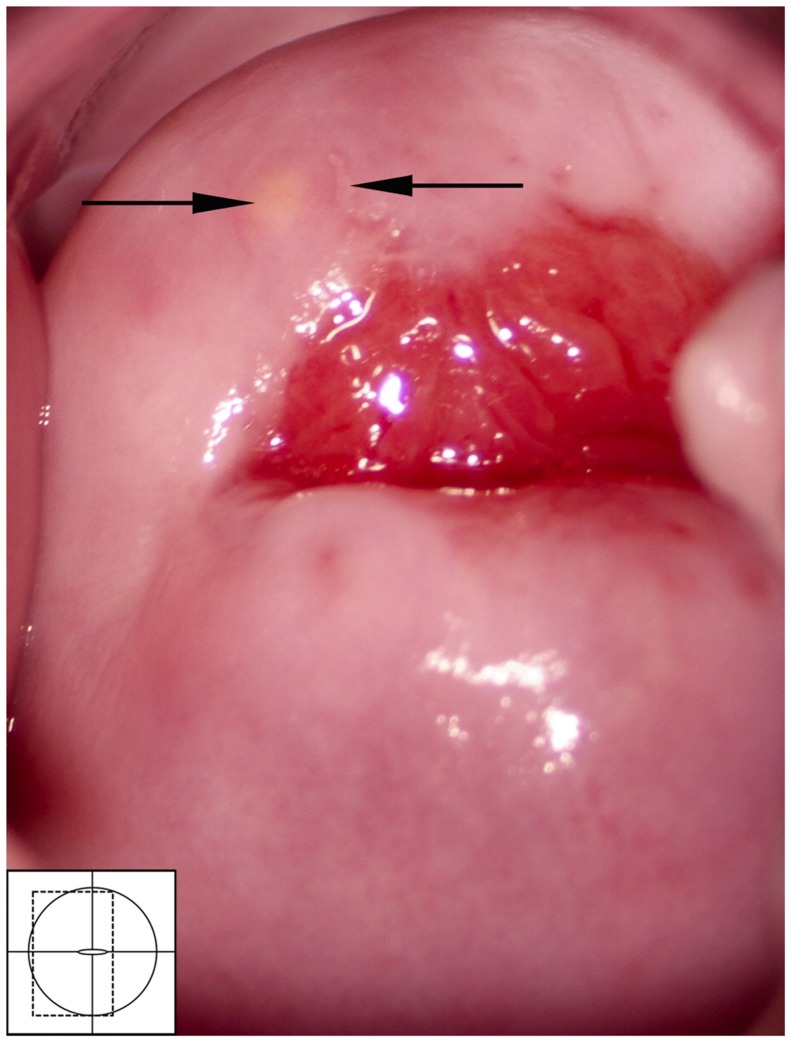
Nabothian cyst. Normal cervical surface with a small yellow Nabothian cyst (arrow) 11 o'clock in the anterior lip of the transformation zone. These may be confused with rubbery papules but the Nabothian cysts are often bigger, do not protrude so acutely, and they are only found in the transformation zone. Rubbery papules, however, may be situated anywhere on the vaginal and cervical surface. Also note, next to the Nabothian cyst (left arrow) a small irregular-shaped leukoplakia area (right arrow) that could be a herpes simplex viral infection.

**Figure 23:**
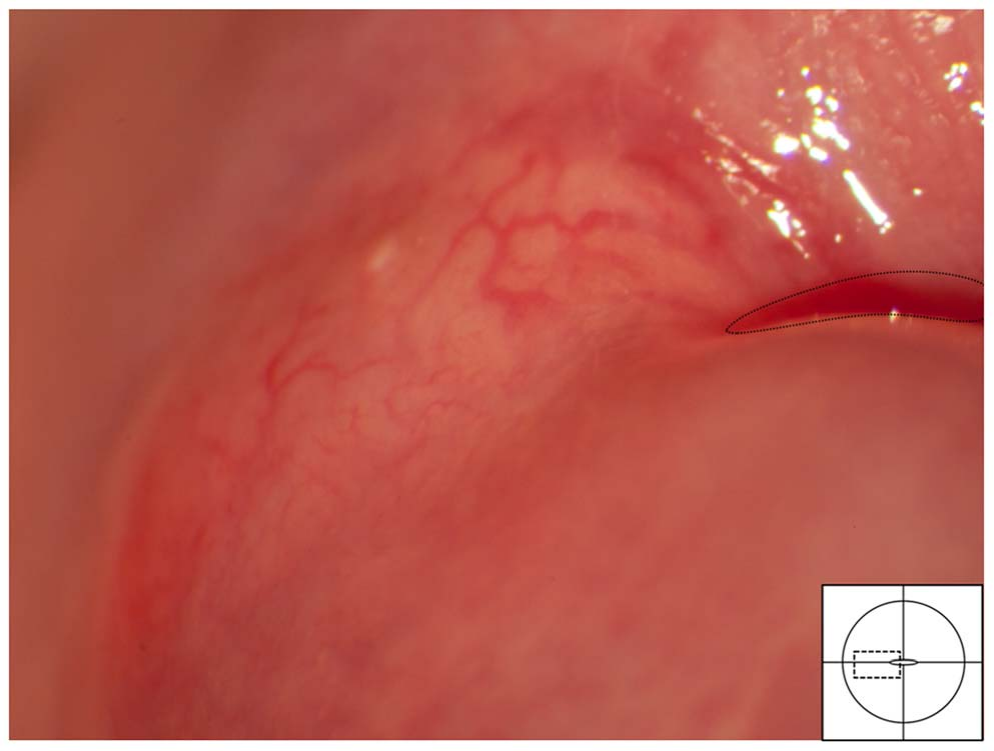
Nabothian cyst. Typical blood vessels across a Nabothian cyst. The underlying cyst is pale yellow adjacent to the squamo-columnar junction (dashed line) and the vascular network shows regular branching.

The normal finding of Nabothian cysts may represent a differential diagnosis to the homogenous sandy patches and rubbery papules (Figures 22, 23). Such cysts represent retention of mucus below the metaplastic squamous epithelium. They are always situated in the transformation zone. They are often single. The shape is circular and there is a central elevation of the mucosal surface. The blood vessels seem to be pushed aside or may cross over the surface.

#### Cervicitis

**Figure 24:**
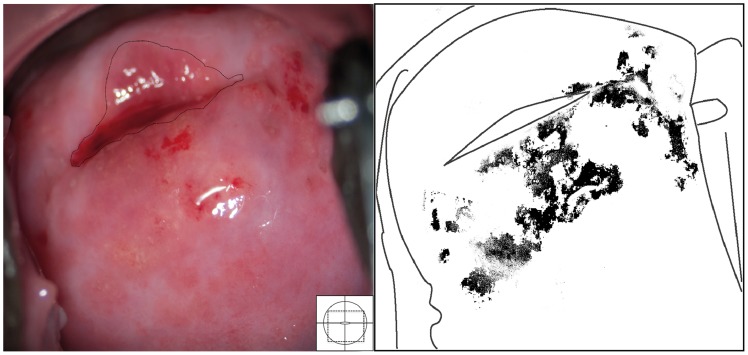
Grainy sandy patches and trichomoniasis together. Clusters of grains on the posterior lip of the ectocervix are both superficial and deep, millimetres to centimetres from the squamo-columnar junction (dashed line). This case is however also positive for *Trichomonas vaginalis* and an erythematous surface is seen with microscopic, punctate haemorrhages typical for trichomoniasis (the so-called ‘strawberry patches’). We also see fresh blood from the mucosal surface. Both diseases may cause such inflammation.

**Figure 25:**
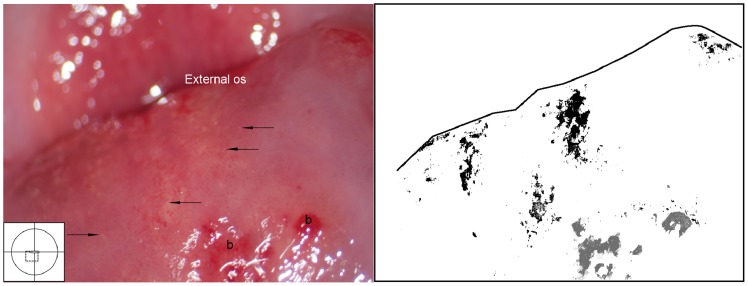
Grainy sandy patches, trichomoniasis, petechiae and mucosal bleeding. We see single grains (arrows point to some examples) scattered over the ectocervical surface. Petechiae are present on the posterior lip. We also see fresh blood from the mucosa (b). The surface is uneven.

Cervicitis is typically characterized by a swollen and hyperaemic cervix and purulent discharge. *S. haematobium* eggs may be found in such cases but are not necessarily the cause (Figure 24, 25). In Trichomoniasis the cervix is strawberry-like, with dilated, often fork-like capillaries (Figure 24). The discharge contains bubbles of gas and one may see mobile flagellates on wet smear.

## Discussion

In schistosomiasis endemic areas, where women have signs of sexually transmitted diseases or malignant-looking lesions, the disease may be female genital schistosomiasis, as presented in these images. Microscopic examination may portray varying degrees of inflammation surrounding the schistosome eggs; from massive accumulation of eosinophils found in the rubbery papules, to moderate immune responses, which may also include CD4 positive lymphocytes and macrophages, and finally fibrous tissue, practically devoid of immune cells [60], [64]. Microscopic examination of the abnormal mucosal blood vessels seen during colposcopy may portray dilated venules or granulation tissue rich in sprouting micro vessels [65]. Thrombosis has also been found to be associated with intravascular schistosome eggs [66].

The four study sites were in the Southern and Eastern regions of Africa. Findings in urinary schistosomiasis studies appear to be relatively similar in the four geographic regions [12], [34]. Likewise, genital sandy patches were similar in all the study sites. Rubbery papules of the genital tract were, however, only seen in Madagascar but have been reported in the urinary tract in Egypt [14], [24]. Likewise, one report in 1962, from South Africa, indicates similar findings [25]. Furthermore, cervices in the Madagascan study site looked similar but were unusually soft. The soft genital tissues made it easy to rotate the speculum for full inspection of the anterior and posterior vaginal surfaces but it was difficult to sample adequate biopsies. To our knowledge this has not been reported previously. The intensity of infection was relatively high in Madagascar, but similar levels were found in Malawi and Zimbabwe [6], [58]. In Madagascar, cytological smears were found to be sensitive and specific indicators of genital schistosomiasis (Randrianasolo, in progress) whereas this has not been the case in other sites. This could indicate differences in the populations' responses to cervical infection. However, we cannot preclude other factors such as differences in epidemiology, e.g. more recent infections, differences in exposure to infested water, genetic and strain differences, or other concomitant diseases [67]. There was one common clinician in all the study sites, investigations were done and images were captured in the same way, group reviews of the photocolposcopic images involved experienced gynaecologists. A review of the older images confirmed the unusual findings in Madagascar.

The studies referred to in this atlas are all epidemiological field studies. We do not know what the lesions look like in pre-pubertal girls or post-menopausal women, since they were excluded from the studies. The cases presented here were likely infected in childhood but there are no clinical studies of the early manifestations of the disease [12], [23], [68]. The colposcopic findings would have been different if done in women seeking medical care for gynaecological symptoms or complaints. The effect of schistosomiasis on conditions such as pelvic organ prolapse, leiomyomas and pregnancies are unknown. Vulvar lesions have not been included in the atlas as none of the clinical community-based studies found that vulvar lesions were associated with urinary or genital *S. haematobium* ova [6], [13], [34], [58]. Secondly, ova can be found in macroscopically normal tissue. In the case reports of vulvar lesions differential diagnostic tests were not done [12]. None of the case reports that found *S. haematobium* in ulcers or tumours presented satisfactory differential diagnostic tests for syphilis, herpes or other possible causes [6], [12], [69], [70]. However, vulvar lesions are less common than other genital symptoms and may require a large sample size to establish a connection. Furthermore, children, unaware of their schistosomiasis status, reported having had more ulcers and genital protuberances if they were positive for urinary schistosomiasis [23]. The findings could not be confirmed by clinical investigation for cultural and ethical reasons. In this atlas, ulcers and tumours have therefore not been presented. Likewise, none of the community-based studies report fistulae. None of the case reports that have found *S. haematobium* in fistulae have performed satisfactory differential diagnostic tests [12]. This aspect has therefore not been included in this atlas.

In many Sub- Saharan African countries, diagnosis for STIs is made syndromically and patients with discharge will be treated for three diseases, *Neisseria gonorrhoeae*, *Chlamydia trachomatis* and *Trichomonas vaginalis*. Without the visual inspection and laboratory analyses, it will be impossible for the clinicians to differentiate FGS from other disease entities [7], [12]. Secondly, STIs and genital schistosomiasis commonly coexist [13], [71]. Thirdly, schistosomiasis in the lower female genital tract may mimic other serious pathology, such as dysplasia and neoplasia. Patients who have been exposed to schistosomiasis are hence at risk of incorrect diagnosis, unnecessary use of antibiotics or surgery, and inadequate treatment. [7], [68], [72].

This overview may provide a platform for increased knowledge about this common disease. The authors hope the atlas will encourage further research into the clinical implications of the disease itself, its implications on fertility and susceptibility to HIV, HPV and other sexually transmitted diseases. If the overview is disseminated beyond the health services for the affluent and the scientific community, it may raise the index of suspicion and may make it possible to diagnose female genital schistosomiasis in rural endemic areas.

**Box 1. Key learning points**

1. The presence of one or more of three aceto-white reaction negative clinical findings may serve as an adequate diagnosis of schistosomiasis in the lower female genital tract for a woman living in an endemic area: sandy patches appearing as (1) single or clustered grains or (2) sandy patches appearing as homogenous, yellow areas or (3) rubbery papules.
2. To diagnose cervicovaginal schistosomiasis all mucosal surfaces must be inspected with an good (non-LED) light source
3. The genital damages have been found to be independent of current water body contact, is acquired in childhood and may increase the risk for other infections, such as HIV [68]
4. Several rounds of anti-schistosomal treatment may be needed to alleviate symptoms. However, clinical findings may persist, and there may be need for invasive, non-pharmaceutical treatment [12].

**Box 2. Interview of the patient**

1. Have you ever, in your lifetime visited a rural/peri-urban area (in tropical or sub-tropical country)? When and where? In these areas do you recall having had contact with fresh water? Did you cross streams to get somewhere? Did you ever fetch water in a river or a lake? Did you go on a boat or fish? Is there any possibility that your tank water was taken from an unsafe water source? Are you sure they used chemicals to clean it?
2. Have you ever had red urine, genital ulcers, swellings/protuberances or genital discharge? Has anyone in your family had this?
3. Have you ever been treated for schistosomiasis/Bilharzia? When and where?

## Supporting Information

Checklist S1 **STROBE checklist.**
(DOC)

Text S1 **Glossary and definitions in this atlas.**
(DOCX)
