# Supplementary material for: The Colposcopic Atlas of Schistosomiasis in the Lower Female Genital Tract Based on Studies in Malawi, Zimbabwe, Madagascar and South Africa
Source: PLoS Negl Trop Dis. 2014 Nov 20;8(11):e3229. doi: 10.1371/journal.pntd.0003229 (PMC4238986; doi:10.1371/journal.pntd.0003229)
Supplement: Text S1 — Glossary and definitions in this atlas. (DOCX) [file pntd.0003229.s002.docx]

# Glossary and definitions in this atlas

Abnormal mucosal blood vessels - convoluted (cork-screw), reticular, circular and/ or branched, uneven-calibered blood vessels visible (by 15 times colposcopic magnification) on the mucosal surface.

Aceto-white reaction negative test (acetic acid test negative or aceto negative) – The cervical epithelium remains unchanged, does not turn white, within 30 seconds after application of 5% acetic acid

CMYK – Cyan-Magenta-Yellow-Black. Recommended colour settings for high-quality print of atlas images

Colposcope – Magnifying instrument used to inspect the mucosal surfaces of the lower female genital tract; usually used in connection with cervical cancer, or suspicion thereof.

Contact bleeding – mucosal bleeding caused by ordinary gynaecological examination or sexual intercourse

Female genital schistosomiasis – One or more of the following findings in women who have been in an *S. haematobium* endemic area: sandy patches appearing as (1) single or clustered grains or (2) sandy patches appearing as homogenous, yellow areas or (3) rubbery papules.

Grainy sandy patches – appearance of single grains, each element resembling a rice grain, measuring approximately 0.05 mm by 0.2 mm, yellow to white in colour, deeply or superficially situated in the mucosa. The lesions are normal by Lugol’s iodine staining, 5% aceto-white reaction negative, and may be situated both within and outside the transformation zone.

Homogenous sandy patches – homogeneous yellow areas without distinct grains when inspected at 15 times colposcopic magnification. The lesions are normal by Lugol’s iodine staining, 5% aceto-white reaction negative, and may be situated both within and outside the transformation zone.

Nabothian cysts – a mucus-filled cyst on the surface of the cervix

Polyp – a smooth pedunculated mass originating from the endocervix or ectocervix

Pre-contact bleeding – darkened blood on the mucosal surface visible upon insertion of the speculum, but not caused by it

Rubbery papules – firm, like a rubber ball, smooth, beige to yellow pustuloid protrusions, measuring 0.3 mm to 1.2 millimetres in diameter. Blood vessels may be identified when micro-focusing with the colposcope; appearing as small spirals under the surface. The lesions are normal by Lugol’s iodine staining, 5% aceto-white reaction negative, and may be situated both within and outside the transformation zone.

Metaplasia – the reversible replacement of one differentiated cell type with another mature differentiated cell type

Transformation zone (TZ) – the area on ectocervix where columnar epithelium has been covered by metaplastic squamous epithelium.

Urogenital schistosomiasis – a term recommended by the World Health Organization (WHO) to replace the term urinary schistosomiasis (*Schistosoma haematobium* infection).
